# ESR Essentials: acute infections of the head and neck—practice recommendations by the European Society of Head and Neck Radiology

**DOI:** 10.1007/s00330-025-11818-4

**Published:** 2025-07-23

**Authors:** Jussi Hirvonen, Ravi Kumar Lingam, Steve Connor

**Affiliations:** 1 Department of Radiology, University of Turku and Turku University Hospital, Turku, Finland; 2 Department of Radiology, Tampere University, Faculty of Medicine and Health Technology, and Tampere University Hospital, Tampere, Finland; 3 Department of Radiology, Northwick Park Hospital, London North West University Healthcare NHS Trust, London, UK; 4 School of Biomedical Engineering and Imaging Sciences, King’s College London, London, UK; 5 Department of Neuroradiology, King’s College Hospital, London, UK; 6 Department of Radiology, Guy’s Hospital and St Thomas’ Hospital, London, UK

**Keywords:** Neck, Infections, Abscess, X-ray computed tomography, Magnetic resonance imaging

## Abstract

**Abstract:**

Acute head and neck infections are common in the population and can have serious complications. Prompt diagnosis and treatment are necessary to avoid morbidity and mortality. Imaging is not indicated for uncomplicated acute rhinosinusitis, otomastoiditis, or limited face and neck soft tissue infections (such as tonsillar or odontogenic infections). However, the presence of facial swelling, severe pain, neurological symptoms, and eye signs suggests a complicated infection and warrants emergency imaging. Contrast-enhanced computed tomography (CT) is the primary imaging modality due to its wide availability and ability to show edema and drainable abscesses in the soft tissues, as well as bone resorption. Contrast-enhanced magnetic resonance imaging (MRI) benefits from superior soft tissue contrast, so it may be additive for the demonstration of orbital, skull base, and intracranial complications, as well as neck soft tissue involvement. Complications, such as orbital or intracranial extension, vascular thrombosis, and mediastinal involvement, should be noted on imaging. Follow-up imaging may be required based on clinical grounds and should mention residual disease and surgical drains.

**Key Points:**

- *In uncomplicated head and neck infections, emergency imaging is not needed.*
- *Contrast-enhanced CT or MRI is indicated in complicated sinus and ear infections.*
- *In neck soft tissue infections, contrast-enhanced CT or MRI is indicated.*

## Key recommendations

- Cross-sectional imaging is not indicated in uncomplicated dental, tonsillar, sinonasal, and middle ear infections (level of evidence: high).
- Contrast-enhanced CT is indicated in the emergency setting for complicated acute sinusitis or middle ear infection and to delineate soft tissue infection, complications, and abscess formation (level of evidence: high).
- Contrast-enhanced MRI has an additional role in the demonstration of orbital, skull base, and intracranial complications and has an evolving role in evaluating soft tissue neck infection (level of evidence: moderate).

## Introduction

Acute head and neck infections are relatively common bacterial diseases that can have serious complications. These infections typically originate from the paranasal sinuses, temporal bone, mouth, throat, salivary glands, lymph nodes, or as a result of post-surgical complications. A common manifestation of a head and neck infection is an abscess, which may require prompt surgical drainage. Complications can be devastating, such as orbital, skull base, or intracranial extension of infection, vascular thrombosis, or descending mediastinitis.

Cross-sectional imaging is typically needed to confirm the diagnosis, evaluate the extent of the infection, detect surgically drainable abscesses, and rule out complications.

This article provides practice recommendations for imaging choices and diagnostic considerations when evaluating acute sinonasal, ear, skull base, face, and neck soft tissue infections. Subacute or chronic infections will not be covered.

## Background

### Sinonasal infections

Sinonasal infections can be viral, bacterial, or fungal, and result from impaired fluid clearance by the mucosa. The clinical criteria include purulent nasal drainage, nasal obstruction, and localized sinus pain or pressure [1]. Sinusitis may also be of odontogenic origin in up to 40% of cases [2]. Acute rhinosinusitis is a clinical diagnosis that does not require imaging [1, 3–5] and should not be suggested solely based on imaging findings of mucosal thickening or fluid collections. Complicated sinusitis is suggested in the presence of severe headache or neck pain, proptosis, decreased vision or diplopia, and facial or orbital swelling, and this warrants prompt imaging [1, 3–6]. The most common complication is orbital spread of infection, while intracranial complications are rare. Imaging studies should investigate the extra-sinus extension of infection, especially into the orbit and the intracranial compartment, and will guide surgical interventions [7]. Orbital complications usually manifest as disease spread from ethmoid sinusitis through the thin medial bony wall of the orbit (lamina papyracea). Orbital abscesses are often located subperiosteally. Orbital and intracranial extension may also occur through neurovascular foramina or valveless veins. Intracranial complications typically include cavernous sinus thrombosis, epidural empyemas, meningitis, cerebritis, and brain abscesses [6].

Acute invasive fungal sinusitis (AIFS) usually results from Zygomycetes/Mucormycetes or invasive Aspergillus species and typically presents in immunocompromised patients with a predisposition to arterial thrombosis [8]. AIFS is a severe disease associated with a high mortality rate (50%).

### Temporal bone and skull base infections

Acute otitis media (AOM) is the most frequent pediatric bacterial infection, with an incidence of 256/1000 person-years [9] as compared to 5.3/1000 person-years in adults [10]. Uncomplicated AOM presents as mild otalgia and middle ear suppuration on otoscopy [11] and does not require imaging even when recurrent. However, imaging is required in cases of complicated AOM, which develops in < 1% of cases [12]. Complicated AOM is characterized by worsening pyrexia, retroauricular pain, and swelling, together with headache and neurological signs [13, 14]. The infection can spread extra-cranially through the mastoid cortex into the post-auricular, temporal, or suprahyoid neck soft tissues or deeply into the inner ear or petrous apex. Intracranial spread may occur through the tegmen tympani to the middle cranial fossa or through the sigmoid plate into the posterior fossa and may be manifest as dural venous sinus thrombosis, extra-axial collections, or intra-axial abscess.

### Face and neck soft tissue infections

Infections of the face and neck soft tissue are typically bacterial and originate from the oral cavity or the throat [15]. Most oral cavity infections are odontogenic, although they may occur due to salivary gland infection. Throat infections are usually secondary to palatine tonsillitis. In children, lymph nodes are often the source of infection. Rarer aetiologies include congenital cysts and sinuses, or tumors.

Facial and neck soft tissue infections may be complicated by abscess formation, which should be demonstrated with imaging. These abscesses may require surgical drainage, especially when large and multicompartmental. Oral cavity infections may result in sublingual, submandibular, buccal, or masticator space abscesses. Odontogenic neck infections are common, especially in the adult population. The disease typically spreads from a periapical infection to the bone, causing acute osteomyelitis, and through the mandibular or maxillary bone into the adjacent soft tissues. Simple tonsillitis does not require imaging, nor do peritonsillar abscesses located between the tonsillar capsule and the superior pharyngeal constrictor muscle. Imaging may be warranted in non-draining peritonsillar abscesses or clinical suspicion of deep space extension to the parapharyngeal or retropharyngeal spaces. In severe neck infections, abscesses may descend into the infrahyoid neck and mediastinum via the visceral, retropharyngeal, and anterior cervical spaces and may result in airway compromise, vascular complications, or septic emboli. Necrotizing fasciitis of the neck is a life-threatening soft tissue infection that may require prompt surgical debridement [16].

## Imaging modalities

### Radiography

Panoramic radiographs of the jaws can assist in identifying the possible source of odontogenic infections; however, they are limited in their ability to visualize soft tissues adequately.

### Ultrasonography (US)

US has high spatial resolution and may be a viable initial imaging method for local facial and neck swelling [17]. The advantage of the US is the possibility of draining and sampling local masses and collections under imaging guidance. The disadvantage is the inability to rule out an extension to deep neck spaces due to the limited penetrance of ultrasounds.

### Computed tomography (CT)

CT is the most commonly used modality for acute head and neck infections due to its widespread availability, speed of acquisition, and reasonable cost [18]. CT protocols for imaging acute complications require intravenous contrast. CT is optimal for the depiction of cortical bony structures and air. Thus, CT is superior to MRI in demonstrating erosion of cortical bony structures in the sinonasal or tympanomastoid region and those severe bacterial infections associated with gas formation. The ability of CT to depict drainable abscesses is based on a low-attenuation fluid collection surrounded by rim enhancement [19]. However, due to the limited soft tissue contrast of CT, differentiating drainable abscesses from non-drainable phlegmon may be cumbersome [20–22]. CT involves ionizing radiation, which needs to be considered, especially in younger patients and when multiple imaging examinations are required to evaluate treatment response.

CT imaging should include thin slices with both soft tissue and bone algorithms and windowing, with reformats in three orthogonal orientations. Intravenous iodine-based contrast agents should be employed and timed for the venous phase. Evaluation of complicated sinonasal infection and AOM will generally require face, skull base, and intracranial coverage, whilst extracranial soft tissue neck infection will be imaged from the skull base to the thoracic inlet. Since descending mediastinitis can be clinically silent, including a part of the upper mediastinum is recommended [15]. Imaging the whole chest with lung and soft tissue reconstructions should be included in patients suspected of descending mediastinitis based on clinical findings of low neck swelling and redness or radiological findings of abscesses reaching the thoracic inlet. Imaging the mediastinum is technically more feasible with CT than MRI.

It should be noted that low mA studies for routine sinonasal and temporal bone imaging are inadequate for demonstrating soft tissue complications.

### Cone-beam computed tomography (CBCT)

CBCT provides high-resolution images from bony structures; however, there is limited depiction of soft tissues due to the low mA. Whereas it may be helpful in general dental, sinonasal, and temporal bone imaging, CBCT is rarely indicated in acute infections since the emphasis is on the depiction of soft tissue complications [23].

### Dual-energy computed tomography (DECT)

Dual-energy or spectral CT offers improved soft tissue sensitivity compared with traditional single-energy CT [24]. These methods are based on CT imaging carried out with two or more types of spectra of X-rays. DECT can more accurately assess the attenuation of material than single-energy CT and can even be used to detect certain materials (such as water, iodine, and calcium). There is preliminary evidence that lower-energy images afforded by these methods improve the delineation of neck soft tissue abscesses [25, 26].

### Magnetic resonance imaging (MRI)

Contrast-enhanced MRI has superior soft tissue contrast and high accuracy in imaging the nature and extent of intracranial, skull base, inner ear, orbital, and neck soft tissue infectious complications. MRI does not involve ionizing radiation, which is helpful in younger patients. MRI accurately delineates normal tissues (e.g., muscles, fat, glandular tissue, lymphoid tissue, and mucosa), detects soft tissue and bone marrow edema, and accurately delineates abscesses [27–29]. Contrast-enhanced MRI shows neck soft tissue abscesses better than CT in a head-to-head comparison [30, 31] and is more sensitive to intracranial complications of acute sinusitis [6, 32]. The disadvantages of MRI include low availability in emergency settings and longer scanning times. In addition, extending the field of view lower to the mediastinum requires more time and effort than when using CT.

Multiplanar imaging is recommended with T2-weighted sequences, with and without fat suppression. T1-weighted imaging should be performed before and after intravenous gadolinium-based contrast agent administration. Fat suppression in post-contrast T1-weighted images will improve the detection of infection-related enhancement. Diffusion-weighted imaging (DWI) with apparent diffusion coefficient (ADC) maps is essential for demonstrating drainable abscesses in the orbit, intracranial compartment, and neck soft tissues. Magnetic resonance venography (MRV) may be indicated in the context of suspected intracranial complications of AOM. In imaging orbital and intracranial complications, thin-section or 3D T1-weighted post-contrast images are recommended.

## Key findings and differential diagnosis

### Sinonasal infections

Acute sinusitis is often associated with opacification of one or more paranasal sinuses, and fluid levels may be seen. Bony structures are rarely destroyed on CT in the acute phase. Whilst there may be increased DWI signal related to purulent contents, this is a non-specific finding, which may simply relate to inspissated secretions.

**Fig. 1:**
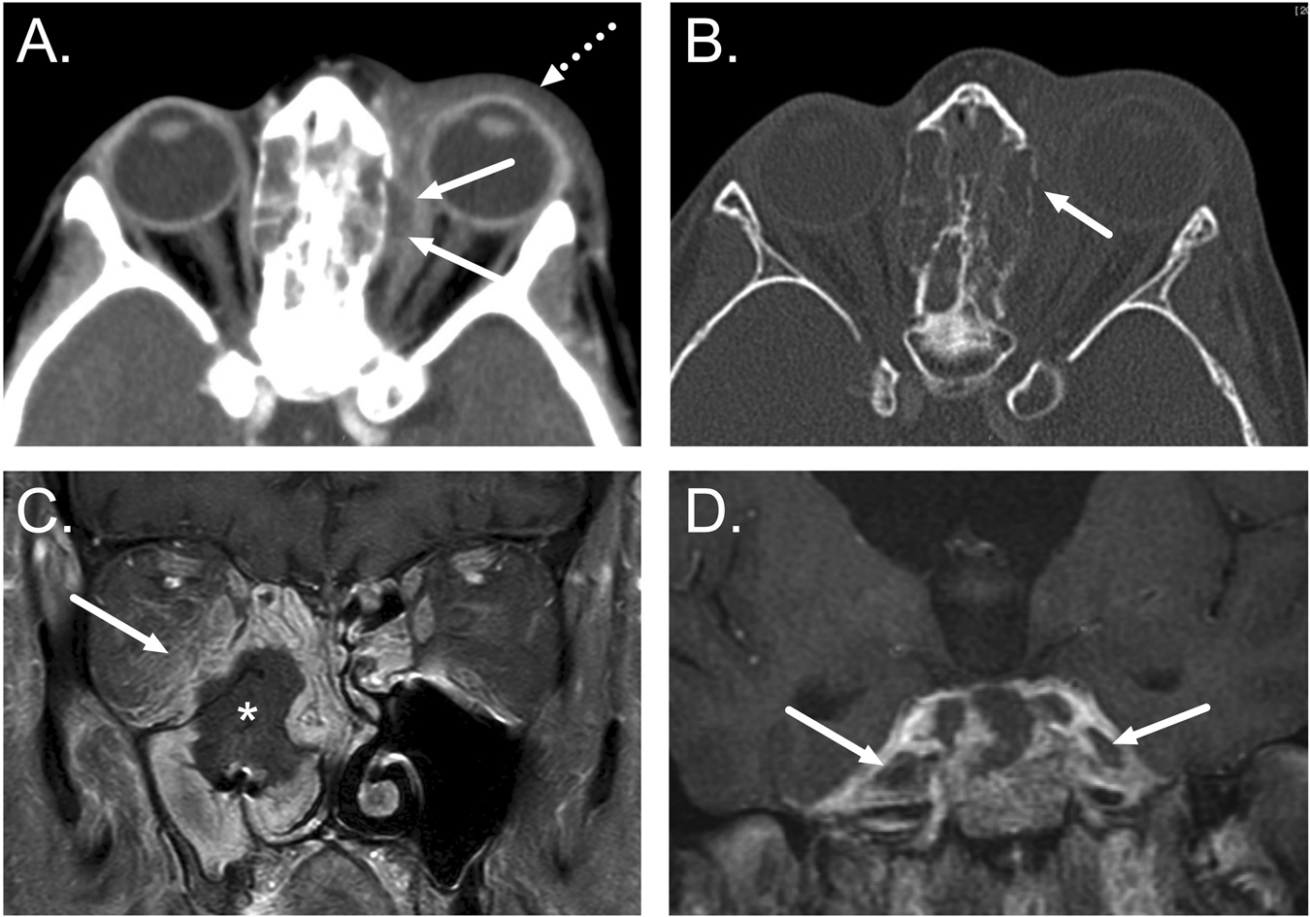
**A**, **B** Complications of acute sinusitis. Axial CT images show a subperiosteal orbital abscess secondary to ethmoiditis (arrows in **A**), with bony dehiscence in lamina papyracea (**B**). Note the preseptal swelling (dotted arrow in **A**). **C**, **D** Aspergillus-related AIFS in an immunocompromised patient. **C** Coronal post-contrast T1-weighted image demonstrates a large non-enhancing area in the right nasal cavity, maxillary sinus, and orbit (“black turbinate sign”, asterisk), along with extensive infective changes in the orbit (arrow). **D** Coronal post-contrast T1-weighted image from another patient shows bilateral cavernous sinus thrombosis as filling defects (arrows)

The spread of infection into the orbit is suggested by preseptal edema (periorbital cellulitis) or postseptal edema (orbital cellulitis). Since the ethmoid sinuses are the most frequent source of orbital extension, they are often opacified. Orbital abscesses are suggested by low attenuation collection on CT images and are often located adjacent to the bone as subperiosteal abscesses (Fig. 1A, B). Pott’s puffy tumor is a rare complication of frontal sinusitis associated with osteomyelitis, destruction of the anterior frontal sinus wall, and a subperiosteal abscess, causing a lump in the forehead.

**Fig. 2:**
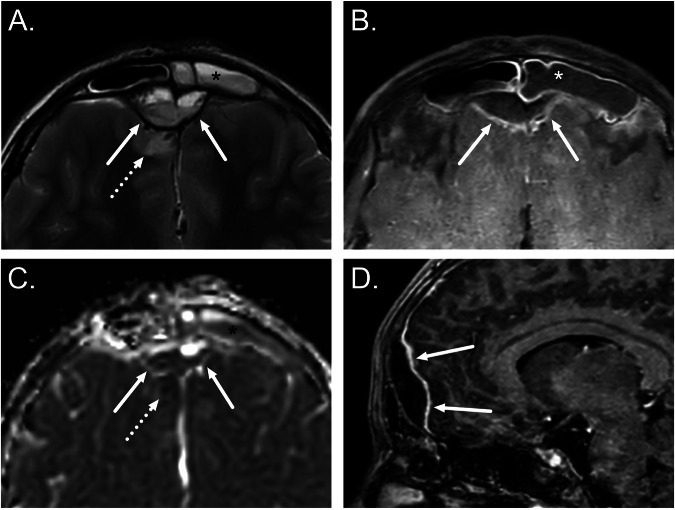
Frontal sinusitis complicated by an epidural abscess, pachymeningitis, and cerebritis. Axial T2-weighted (**A**), axial fat-suppressed post-contrast T1-weighted (**B**), and sagittal post-contrast T1-weighted (**D**) images and an axial ADC map (**C**) demonstrate purulent fluid in the left frontal sinus (asterisks) and an epidural purulent collection with dural enhancement (arrows). Edema (**A**) and restricted diffusion (**C**) in the adjacent brain tissue (dotted arrows) suggest cerebritis

Intracranial complications of acute sinonasal infections include extra-axial empyemas, meningitis, cerebritis, and brain abscesses (Fig. 2). Epidural empyemas are most often secondary to frontal sinusitis, and the posterior wall of the frontal sinus may remain intact when there is spread through valveless venous structures (veins of Breschet) [33]. Filling defects on contrast-enhanced CT or MRI in the cavernous sinus suggest thrombosis.

AIFS is suggested by a lack of mucosal enhancement (“black turbinate” sign), bony destruction, and extra-sinus extension (Fig. 1C, D). The spread of infection to the adjacent facial soft tissues is more frequent with acute fungal sinusitis and is suggested by soft tissue thickening or edema within the pre-maxillary or retro-maxillary fat planes. In addition to orbital complications, AIFS is characteristically angio-invasive, resulting in aneurysm formation and cerebral infarction.

### Temporal bone and skull base infections

**Fig. 3:**
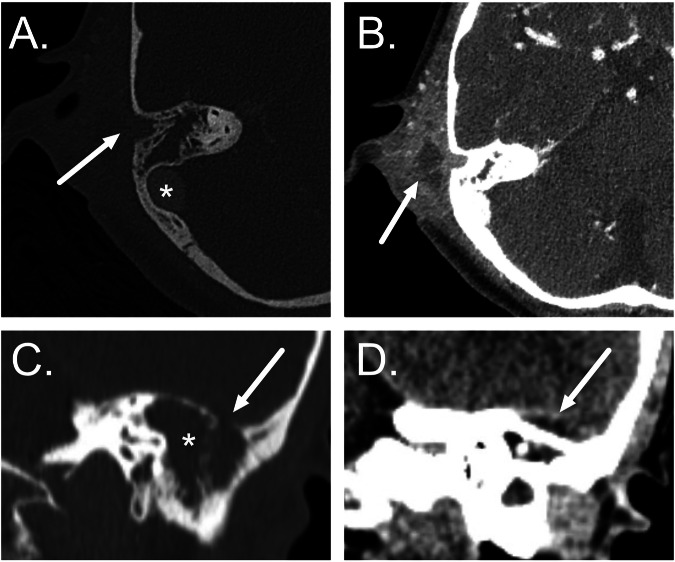
Acute coalescent mastoiditis. **A**, **B** Axial post-contrast CT images show localized erosion of the mastoid septae and lateral bony cortex (arrow in **A**) by acute coalescent mastoiditis associated with an adjacent post-auricular inflammatory abscess (arrow in **B**). Note the normally enhancing sigmoid sinus (asterisk in **A**) adjacent to the intact bony sigmoid plate. **C**, **D** Coronal contrast-enhanced CT images show opacified mastoid with septae destruction (asterisk in **C**) and a focal bony erosion at the tegmen mastoid (arrow in **C**) with an adjacent extradural abscess (arrow in **D**)

AOM can be complicated by coalescent mastoiditis [34], which is characterized by progressive bony resorption and destruction of mastoid septae and cortices on CT (Fig. 3). The most specific imaging feature is the erosion of the sigmoid plate. Normal asymmetry in the configuration of the opacified mastoid air cells, cortical defects due to arachnoid granulations, and retrosigmoid accessory emissary veins should not be misinterpreted as bony erosion. In addition, it should be noted that ”subclinical” mastoid opacification is frequently demonstrated in the context of AOM and is of little significance in isolation. Contrast-enhanced MRI can separate purulence from simple effusion by intra-mastoid lowered T2 signal intensity, enhancement, and restricted diffusion [35]. The possibility of an infected cholesteatoma should be considered in the context of increased DWI signal in the middle ear and mastoid.

Extracranial spread of mastoid infection may occur through cortical erosion or via a transvenous route (Fig. 3). Extension is most frequently demonstrated directly laterally into the post-auricular region, with inferior extension from the mastoid tip (Bezold’s abscess), superior extension to the temporal fossa (Luc’s abscess), posterior extension (Citelli’s abscess) and anterior extension to the zygomatic root also being possible.

**Fig. 4:**
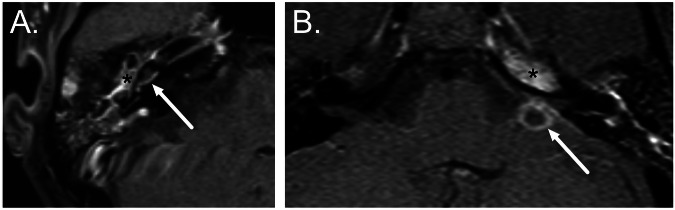
Medial extension of AOM on axial post-contrast T1-weighted MRI. **A** Acute labyrinthitis: AOM with inflammatory enhancement in the middle ear cleft (asterisk) associated with abnormal enhancement in the cochlea (arrow) indicating labyrinthitis. **B** Petrous apicitis with Gradenigo syndrome: AOM with inflammatory enhancement extending medially into the petrous apex (asterisk) with the development of an intracranial abscess adjacent to the petrous ridge (arrow)

Medial extension of infection from the middle ear may result in tympanogenic labyrinthitis (Fig. 4A), whilst perilabyrinthine air cells may act as a conduit to the petrous apex (Fig. 4B). Whereas CT may demonstrate cortical bone erosion and air cell opacification in petrous apicitis, involvement of the cancellous bone and any extra-axial extension is best depicted with MRI [34]. Patients with petrous apicitis are generally febrile and unwell with some or all features of the Gradenigo triad (ipsilateral abducens nerve palsy, otalgia, and facial pain) [36] (Fig. 4B).

### Neck soft tissue infections

Patients with neck soft tissue infections may have airway compromise and unstable haemodynamics if severely ill. These patients will need to be stabilized and have their airway secured before cross-sectional imaging.

Infection of the neck soft tissues is suggested by tissue edema, manifesting as fat stranding and fascial thickening on CT and high signal intensity areas on fat-suppressed T2-weighted MR images [29]. Edematous and enhancing tissue is referred to as cellulitis. Due to its superior soft tissue contrast, contrast-enhanced MRI can demonstrate edema patterns that predict a severe course of illness [28].

The CT criteria for abscesses include mass effect, central hypodensity, and rim enhancement [19]. A limitation of CT in distinguishing drainable abscesses from non-drainable phlegmons [20]. On MRI, abscesses present as collections with hyper- or isointense T2-signal intensity, no enhancement on post-contrast T1-weighted images, and restricted diffusion on DWI (low ADC values) [27]. Necrotic, poorly enhancing lymph nodes may mimic suppurative lymphadenitis (intranodal abscesses) and result in false-positive findings [27]. The US can detect abscesses as hypoechoic collections [17] and assess the neck veins to rule out thrombosis.

**Fig. 5:**
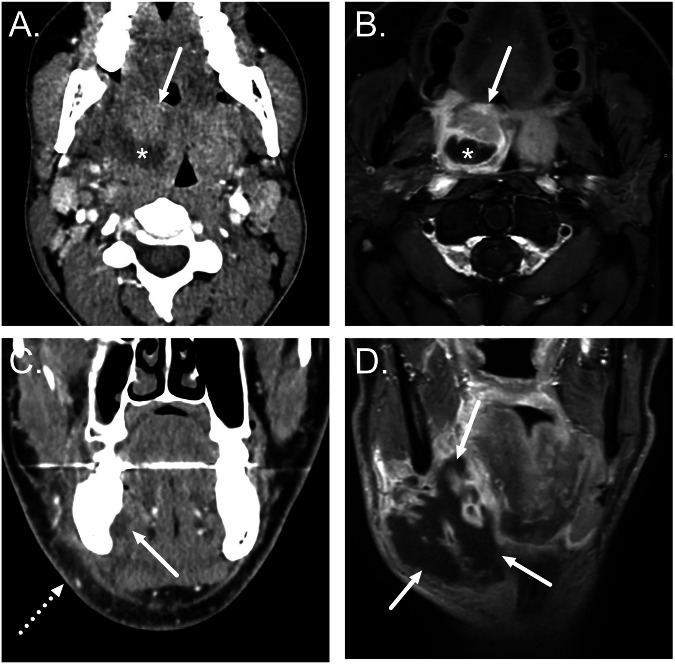
**A**, **B** Examples of peritonsillar abscesses. **A** Axial CT image shows a hypodense collection (asterisk) behind the right palatine tonsil (arrow). **B** Axial fat-suppressed post-contrast T1-weighted image shows a non-enhancing hypointense collection (asterisk) behind the right palatine tonsil (arrow). **C**, **D** Examples of odontogenic neck abscesses. **C** A coronal CT image shows a hypodense collection (arrow) medial to the mandible, consistent with a subperiosteal abscess. Note the subcutaneous fat stranding (dotted arrow). **D** A coronal fat-suppressed post-contrast T1-weighted image shows a large multilocular abscess extending from the submandibular space to the anterior cervical space

Tonsillitis appears as tonsillar and peritonsillar enhancement with tonsillar non-enhancing thin fluid collection (“tiger stripe” appearance). Peritonsillar abscesses can be seen between the tonsillar capsule and the superior constrictor muscle [37] (Fig. 5A, B). They can cross the superior constrictor muscle and the buccopharyngeal fascia posteriorly into the retropharyngeal space and laterally to the parapharyngeal and carotid spaces [37]. From there, they may extend caudally into the submandibular and visceral spaces and the infrahyoid neck.

Odontogenic infections originating from mandibular teeth may spread to the sublingual (anterior teeth) and submandibular spaces (posterior molars) (Fig. 5C, D). Severe cellulitis and abscess formation in the floor of the mouth is called Ludwig’s angina. Maxillary odontogenic infections can spread to the buccal and masticator spaces and even to the parapharyngeal space from the posterior molars. In sialadenitis (salivary gland infection), the salivary glands appear edematous. Calculi and dilated ducts may be seen in obstructive sialadenitis [38].

Infected lymph nodes, or suppurative lymphadenitis (intranodal abscesses), are typically present in children, either in lateral neck lymph nodes or the lateral retropharyngeal space. Suppurative lymphadenitis is suggested by an enlarged lymph node with a low-density center on CT or non-enhancement and low ADC values on MRI. A differential diagnostic consideration is necrotic lymphadenitis with slower enhancement but no suppuration [27]. Retropharyngeal suppurative lymph nodes can rupture and spread into the retropharyngeal space between the fascial sheets.

**Fig. 6:**
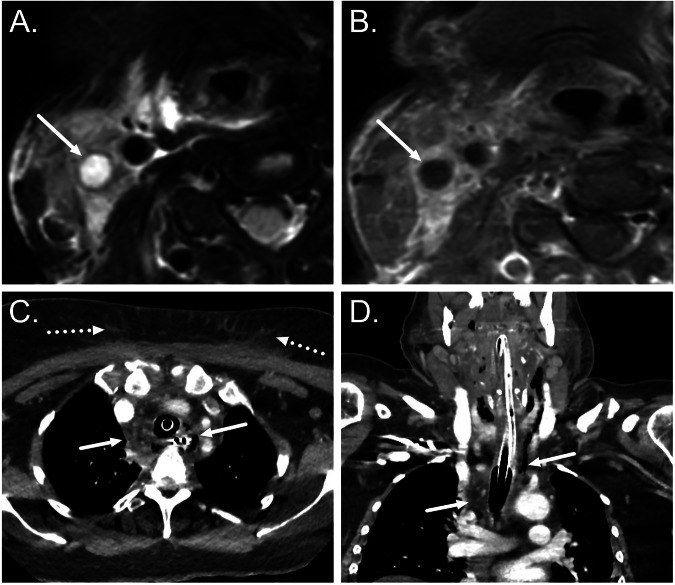
Complications of neck soft tissue infections. **A**, **B** Internal jugular vein thrombosis. Axial fat-suppressed T2-weighted (**A**) and post-contrast T1-weighted (**B**) MR images show a thrombosed internal jugular vein (arrows). **C**, **D** Descending mediastinitis. Axial (**C**) and coronal (**D**) contrast-enhanced CT images show edema and gas formation in the mediastinum (arrow). Note the subcutaneous fat stranding (dotted arrows) and the endotracheal and nasogastric tubes. Image partly adopted from the reference [29] with permission under the Creative Commons Attribution 4.0 International License

Lemierre’s syndrome is a complication of a bacterial neck infection and is characterized by internal jugular vein thrombosis and septic lung emboli [39] (Fig. 6A, B). Necrotizing fasciitis is suggested by widespread tissue edema, fluid collections along the fascial planes, and gas formation [16].

Descending mediastinitis is characterized by the spread of edema and/or abscess from the infrahyoid anterior neck spaces (visceral and anterior cervical spaces) or directly from the retropharyngeal space [40] (Fig. 6C, D).

Important differential diagnostic considerations in the neck soft tissues include non-infective inflammatory conditions, such as longus colli calcific tendonitis, carotidynia, and angioedema [29].

## Follow-up imaging

Imaging after surgery may be warranted to diagnose residual or non-drained abscesses and to confirm the position of surgical drains. Presurgical images help verify the status of the abscess, although smaller size, postoperative edema, and surgical drains may hinder this evaluation. Surgical drains are easily seen with CT and MRI [29].

## Summary statement

**Fig. 7:**
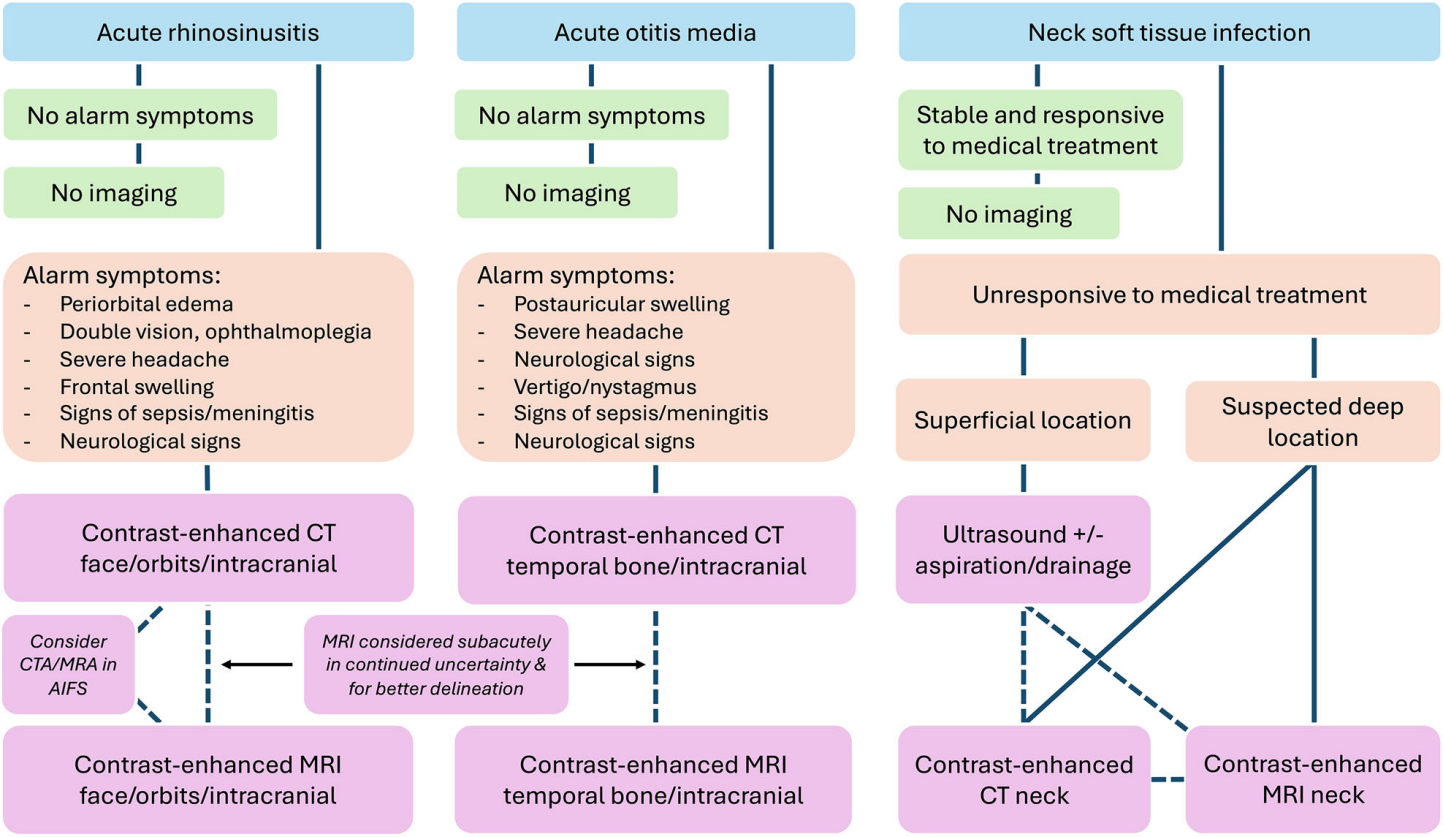
Flowchart for imaging acute head and neck infections. Solid lines denote the suggested pathway, and dashed lines denote optional approaches

Cross-sectional imaging is critical in diagnosing and managing acute head and neck infections (Fig. 7). While uncomplicated infections are managed based on clinical grounds, suspicion of infection spread to adjacent soft tissue spaces warrants cross-sectional imaging to rule out complications. Complicated sinonasal infection may spread to the orbit or intracranially, whilst middle ear infection can spread to the extracranial soft tissues, the inner ear, the petrous apex, and intracranially. In these scenarios, CT with contrast is indicated in the emergency setting, with contrast-enhanced MRI having an additional role due to improved soft tissue characterization. In infections of the neck soft tissue spaces, such as those originating from the pharynx or the oral cavity, US may be considered when superficial infections are suspected. In suspected deep infections, CT with contrast is the most commonly used emergency imaging modality to demonstrate drainable abscesses and rule out complications. In this setting, contrast-enhanced MRI has an evolving role due to improved soft tissue contrast, but lower availability limits its use. The US may be used for superficial infections. In severe soft tissue infections, imaging the chest may be warranted to rule out descending mediastinitis, CT being more feasible than MRI in this regard. Follow-up imaging may be requested to rule out residual abscess cavities and confirm the placement of surgical drains.

## Patient summary

While limited infections can be diagnosed based on patient examination, severe acute infections may need medical imaging for diagnosis and management. These severe infections can spread from the paranasal sinuses, mouth, or ear into the eye, brain, or neck soft tissues. CT is usually the preferred method to image these infections in the first instance. MRI is sometimes needed because it can better demonstrate different tissues. Both CT and MRI are performed using an intravenous contrast agent. Imaging will confirm the diagnosis, assess complications, provide an anatomical map for the surgeons, and evaluate treatment response in follow-up when needed.

## Abbreviations

ADC: Apparent diffusion coefficient
AIFS: Acute invasive fungal sinusitis
AOM: Acute otitis media
CBCT: Cone-beam computed tomography
DWI: Diffusion-weighted imaging
